# Clinical acceptability of automatically generated lymph node levels and structures of deglutition and mastication for head and neck radiation therapy

**DOI:** 10.1016/j.phro.2024.100540

**Published:** 2024-02-01

**Authors:** Sean Maroongroge, Abdallah SR. Mohamed, Callistus Nguyen, Jean Guma De la Vega, Steven J. Frank, Adam S. Garden, Brandon G. Gunn, Anna Lee, Lauren Mayo, Amy Moreno, William H. Morrison, Jack Phan, Michael T. Spiotto, Laurence E. Court, Clifton D. Fuller, David I. Rosenthal, Tucker J. Netherton

**Affiliations:** a Department of Radiation Oncology, Division of Radiation Oncology, University of Texas MD Anderson Cancer Center, United States; b Department of Radiation Physics, Division of Radiation Oncology, University of Texas MD Anderson Cancer Center, United States

**Keywords:** Deep learning, Segmentation, Chewing and swallowing structures, Lymph node levels, Radiotherapy, Contouring

## Abstract

**Background and Purpose:**

Auto-contouring of complex anatomy in computed tomography (CT) scans is a highly anticipated solution to many problems in radiotherapy. In this study, artificial intelligence (AI)-based auto-contouring models were clinically validated for lymph node levels and structures of swallowing and chewing in the head and neck.

**Materials and Methods:**

CT scans of 145 head and neck radiotherapy patients were retrospectively curated. One cohort (n = 47) was used to analyze seven lymph node levels and the other (n = 98) used to analyze 17 swallowing and chewing structures. Separate nnUnet models were trained and validated using the separate cohorts. For the lymph node levels, preference and clinical acceptability of AI vs human contours were scored. For the swallowing and chewing structures, clinical acceptability was scored. Quantitative analyses of the test sets were performed for AI vs human contours for all structures using overlap and distance metrics.

**Results:**

Median Dice Similarity Coefficient ranged from 0.77 to 0.89 for lymph node levels and 0.86 to 0.96 for chewing and swallowing structures. The AI contours were superior to or equally preferred to the manual contours at rates ranging from 75% to 91%; there was not a significant difference in clinical acceptability for nodal levels I-V for manual versus AI contours. Across all AI-generated lymph node level contours, 92% were rated as usable with stylistic to no edits. Of the 340 contours in the chewing and swallowing cohort, 4% required minor edits.

**Conclusions:**

An accurate approach was developed to auto-contour lymph node levels and chewing and swallowing structures on CT images for patients with intact nodal anatomy. Only a small portion of test set auto-contours required minor edits.

## 1 Introduction

Artificial intelligence (AI) based auto-segmentation models are being adopted in clinical practice within radiation oncology. The benefits of such approaches are well known and include time-savings [1], [2], decreases in variability [3], and quality assurance applications [4], [5], [6]. Such advances are pertinent to the head and neck, as delineation accuracy of OARs (organs at risk) and targets are limited by interobserver and trial protocol variability [7], [8], [9]. Within the anatomic site of head and neck, auto-segmentation models developed through deep learning has resulted in a myriad of contouring approaches [2], [4], [10], [11], [12], [13], [14], [15], [16], [17], [18], [19]. Many of these models focus on contouring OARs that can be delineated from radiotherapy simulation computed tomography (CT) scans. Commercial models for fully automated target segmentation in the head and neck are not yet available, but research in this area is gaining momentum.

Of recent interest is the auto-segmentation of the low-risk, or elective clinical target volume (CTV). The low-risk CTV is comprised of anatomically-defined lymph node-containing regions (“lymph node levels”) that are at risk of metastatic spread, though possess no clinical or radiographic evidence of disease at the time of treatment. The set of lymph node levels selected for inclusion in the low-risk CTV is based on lymphatic drainage patterns from the location of the primary tumor. Generally accepted volumes based on common tumor locations are well documented in consensus guidelines and contouring resources for the head and neck [9], [20]. Automatic CT-based segmentation of these lymph node levels (e.g. I-V) is achievable and has been demonstrated by numerous works [10], [21], [22], [23], [24], [25], [26], [27]. Our clinic previously integrated a deep-learning based approach to contour elective lymph node levels in CT scans [10]. This approach groups nodal levels into families (e.g. IA-V, IB-V, II-IV, and retropharyngeal [RP]) so that elective CTVs can be quickly constructed using Boolean algebra for use in manual and automatic treatment planning. However, one significant challenge in the field of target segmentation is keeping pace with changes in clinical practice. Sources of such changes can be driven by changes in contouring guidelines, improvements in image-guidance technology, or evolving evidence that alters our understanding of the balance between toxicity and tumor control [28], [29]. Strijbis et also noted in their work on automated segmentation of levels I-V that contours produced by Cardenas et al., are generous, resembling their institution’s PTVs [27]. Based on physician feedback and changes in clinical practice, we sought to develop a new auto-segmentation model that more accurately reflects the narrower treatment volumes utilized in our clinic’s practice today.

Proximal to these CTVs are essential structures which enable deglutition (i.e. swallowing) and mastication (i.e. chewing). Although publicly available models and repositories exist for OARs in the head and neck, to our knowledge there exists no such repository of swallowing structures. Teguh et al., were the last to clinically validate an approach to segment lymph levels and swallowing structures in the head and neck in one combined work, but did so with atlas-based auto-segmentation [21]. Many other authors have studied swallowing and chewing structure segmentation, but only include some of the major swallowing and chewing structures that can be visualized on CT [21], [30], [31], [32]. A comprehensive auto-contouring approach to generate these structures could provide a means for reliable and efficient assessment of dose response studies—especially since manual delineation of these many structures is extremely labor intensive.

The purpose of this work was to present a straightforward approach that can efficiently and effectively create and validate a clinical segmentation tool for 1) individual lymph node levels and 2) swallowing and chewing structures in the head and neck. For the lymph node levels, the hypothesis of this work is AI contours will be clinically acceptable and be preferred at rates equal or superior to manual contours. Such an auto-contouring model could eliminate sources of intra-physician variability present in elective target contouring if used clinically. This was tested using two double blinded studies to score physician preference and contour quality (for physician vs AI contours). For the swallowing structures, the clinical acceptability of resulting AI contours was scored by a physician.

## 2 Materials and methods

### 2.1 Patient data

A total of 145 patients who underwent radiotherapy treatment for oropharyngeal cancer at MD Anderson Cancer Center from 2012 to 2020 were retrospectively analyzed under a protocol approved by an Institutional Review Board. The criteria for inclusion in this protocol were: a confirmed pathological diagnosis of oropharyngeal squamous cell carcinoma and (2) patient must have received intensity modulated radiotherapy. The data set of 145 patients were composed of two separate cohorts; one cohort was collected for the lymph node levels and the other for the swallowing and chewing structures. Forty-seven patients were in the lymph node levels cohort. These patients received definitive radiotherapy to the oropharynx with no nodal dissection. The remaining 98 patients formed the swallowing/chewing structures cohort.

### 2.2 Segmentation of ground truth contours

On CTs from 15 patients, lymph node levels (IA, IB, II, III, IV, V, RP) were manually contoured by five radiation oncologists. Each radiation oncologist contoured three different patients. Contours (7 levels x 15 patients = 105 total) were anatomically drawn without margin according to institutional practice and constituted the testing dataset for the lymph node level segmentation model.

One radiation oncologist contoured 17 structures involved in swallowing and chewing in the head and neck (tongue, thyroid cartilage, cricoid, cricopharyngeus, glottic area, supraglottic larynx, buccinators, inferior constrictor, medial constrictor, superior constrictor, anterior digastric, posterior digastric, genioglossus, masseter, mylogeniohyoid (mgh) complex, lateral pterygoid, and medial pterygoid) on 20 patients. This cohort was used as the swallowing/chewing structures testing dataset.

### 2.3 Training and testing methodologies

A three-step methodology was used to create the final multi-class lymph node level segmentation model. First, the publicly available nnUnet tool was used to train a multiclass segmentation model using the small, testing dataset of 15 patients (defined above) [33]. A 3D full resolution model was used with five-fold cross validation; all augmentations were enabled; random initialization was used. Left and right contours for each nodal level were combined into one volume to prevent misclassification from left-right flipping augmentations. Second, the model was used to generate lymph node level contours on 32 additional patients (described above) and were edited by a radiation oncology resident. Third, the final model was trained from scratch with the 32 patients and tested on the original cohort of 15 patients. The ensembled result from the 5 folds was used in the inference stage.

A similar three-step methodology was used to create the swallowing/chewing structures model. First, 20 patients’ structures were manually contoured for use as a standard, and an atlas-based model using Elekta Admire (Elekta AB, Stockholm, Sweden) with batch fusion was created. Second, the atlas was run on the remaining 78 and manually revised by a radiation oncologist. Third, two models were trained to accommodate GPU memory (one with 10 structures and another with 7 structures). The data from the manually revised 78 patients were used for training, and the 20 remaining patients were used for testing. The nnUnet settings to train the models were the same as those mentioned above.

### 2.4 Quantitative and qualitative analyses

This work followed the recommendations by Baroudi et al., which suggest guidance for quantitative (using overlap and distance metrics) and qualitative evaluation (using physician review) of clinical acceptability [34]. Dice Similarity Coefficient (DSC) [35] and 95th percentile Hausdorff (HD95th) distance were used as quantitative performance metrics between the ground truth contours and the predicted contours.

Two blinded studies were performed. In the first blinded study, physicians viewed AI and manual contours and indicated preference for ‘AI’, ‘manual contour’, ‘either', or ‘neither’ in a scoring rubric. There were 525 scores collected in the lymph node contour preference dataset (5 physicians x 15 patients x 7 contours). A period of greater than 6 months took place between initial contouring and this blinded study. Physician scores of preference reflect the frequency of whether their own manual contours, the AI contours, or a colleague’s contours are preferred.

The second blinded study used the same 15 patient cohort. The goal of this blinded study was to quantify the degree to which contours require editing before clinical use. Quantitative scores and comments for each contour (AI and manual) were collected for these patients. Due to the labor-intensive nature of this task, each physician only rated 3 patients each. One of the three patients was originally contoured by the scoring physician; two of the three patients were originally contoured by a different physician. A 5-point Likert scale was used from Baroudi et al., [34].

Student’s t-tests were used to quantify whether the mean of the clinical acceptability scores were significantly different (p < 0.05) for manually generated versus AI generated contours. The aforementioned five-point Likert scale was used to qualitatively score structure predictions on the 20-patient test set from the swallowing/chewing structures patient cohort. Clinically acceptability was scored by the same physician that curated the ground truth dataset; a period of 6 months took place between curation and testing.

## 3 Results

**Fig. 1:**
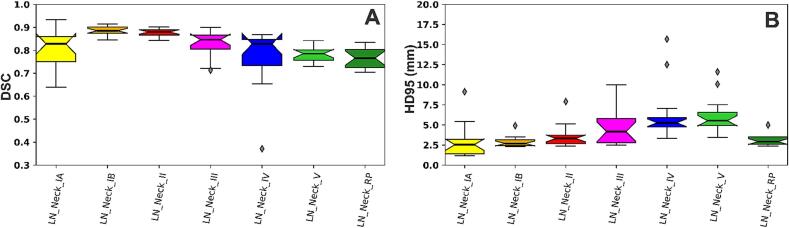
Quantitative metrics for lymph node level segmentation. dsc, dice similarity coefficient; HD95, 95th percentile Hausdorff distance.

Median DSC (Fig. 1A) for the test set (n = 15) were 0.83 (IA), 0.89 (IB), 0.88 (II), 0.85 (III), 0.83 (IV), 0.79 (V), 0.77 (RP). Median HD95^th^ (Fig. 1B) were 2.5 (IA), 2.7 (IB), 3.3 (II), 4.2 (III), 5.3 (IV), 5.5 (V), and 2.9 (RP) millimeters.

**Fig. 2:**
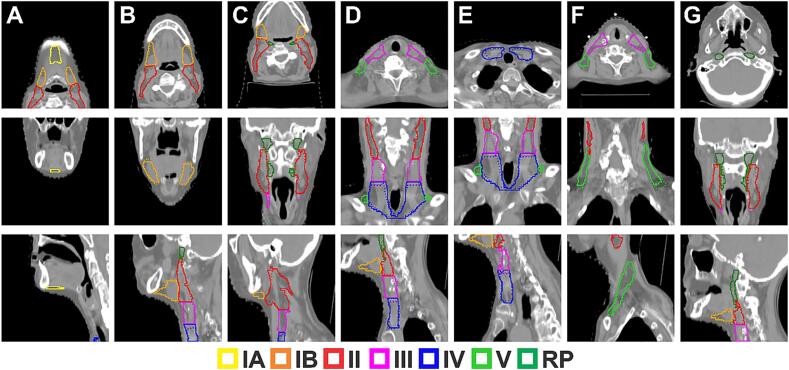
Segmentations of lymph node levels IA, IB, II, III, IV, V, and RP (columns A-G). Solid lines represent ground truth segmentations and dotted lines represent predicted segmentations.

Across all contours, physicians preferred the AI contours at a rate of 247/525 = 47 %. The manual contours were preferred at a rate of 88/525 = 17 %. The AI contour was superior to or equally preferred to the manual contour at a rate of 436/525 = 83 %. This rate is the sum of “AI” and “Either” (Table 1). For levels IA, IB, II, III, IV, V, and RP, the AI contour was superior to or equally preferred to the manual contour at rates ranging from 75 % to 91 %. One physician scored only 1 of 525 contours (level IV) as preferring neither the AI nor the manual contour. In this instance, the physician that made the ground truth manual contour and the physician that scored were different physicians. On average, physicians preferred their original contours (when unknowingly reviewing their own contours which were blinded) over that of the AI contours only 18 % of the time (range = 0–29 %). See (Fig. 2).

**Table 1:** Physician preference scoring for manual and AI contours.

	IA	IB	II	III	IV	V	RP	Sum
Either	60 %	45 %	29 %	44 %	28 %	19 %	27 %	36 %
AI	24 %	40 %	51 %	44 %	47 %	60 %	64 %	47 %
Manual	16 %	15 %	20 %	12 %	24 %	21 %	9 %	17 %
Neither	0 %	0 %	0 %	0 %	1 %	0 %	0 %	0 %
AI or Either	84 %	85 %	80 %	88 %	75 %	79 %	91 %	83 %

In a blinded Likert scale assessment of each nodal level, scores for the manual vs AI contour were significantly different for only the RP contours (p < 0.01), for which AI was higher (Table 2). Thus, there was not a significant difference in clinical acceptability for nodal levels I-V for manual versus AI contours. When radiation oncologists scored their own or other radiation oncologists’ contours, no edits were required (score of 4 or 5) for 82 % of contours. When radiation oncologists scored AI contours, no edits were required for 92 % of contours.

**Table 2:** Physician scoring of clinical acceptability for manual and AI contours.

	IA	IB	II	III	IV	V	RP	All
AI	4.2 (3–5)	4.4 (4–5)	4.2 (3–5)	4.4 (4–5)	4.1 (3–5)	4.1 (3–5)	4.5 (3–5)	4.3 (3–5)
Manual	4.4 (3–5)	4 (3–5)	3.9 (3–5)	4.1 (3–5)	3.9 (3–5)	3.9 (2–5)	3.7 (3–4)	3.9 (2–5)
p-value	0.37	0.07	0.06	0.11	0.42	0.42	<0.01	<0.01

**Fig. 3:**
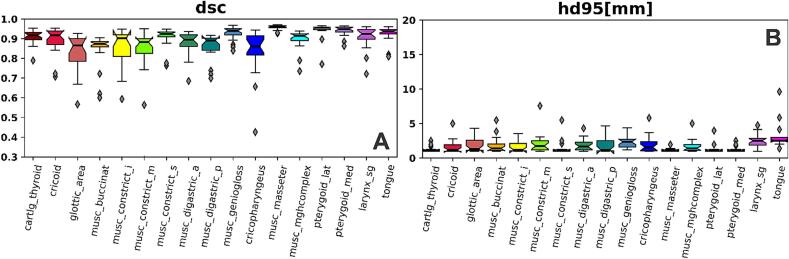
Quantitative metrics for swallowing/chewing structure segmentations. dsc, dice similarity coefficient; hd95, 95th percentile Hausdorff distance.

**Fig. 4:**
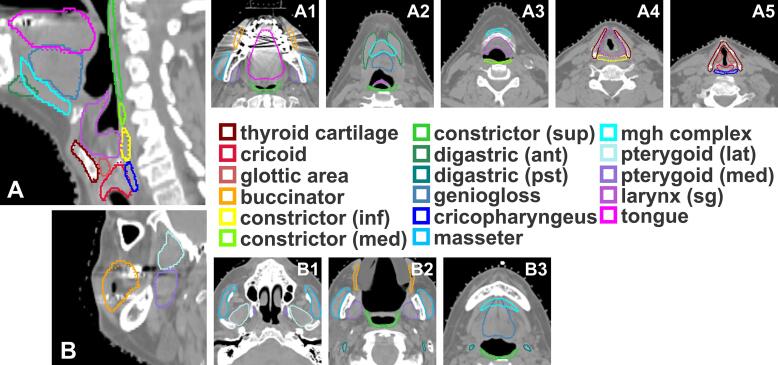
Segmentations of all swallowing/chewing structures. 5A is the mid-sagittal plane that cuts through a majority of the midline structures; 5B is a sagittal plane that cuts through the buccinator and pterygoids. Solid lines represent ground truth segmentations and dotted lines represent predicted segmentations. In many of the figures, solid and dotted lines are overlapped.

Median DSC for the test set (n = 20) ranged from 0.86 to 0.96 (Fig. 3A); median HD95^th^ (in mm) ranged from 1.2 to 2.5 (Fig. 3B). Median DSC was greater than 0.90 for 11/17 structures. Fig. 4 depicts ground truth and AI segmentations for all structures.

Of the 340 contours reviewed, 100 % were found to be 3 or greater (minor edits - use as is). Overall, 96 % percent of contours were scored as 4 or 5 (stylistic differences, use-as-is). Thyroid cartilage, glottic area, larynx, buccinators, digastrics, masseters, mgh complex, and pterygoids had perfect scores (5′s) across all patients. Four common issues were identified (1) the genioglossus would contour into the mgh complex when transitioning, (2) the anterior digastrics overcontour into the mgh complex, (3) the anterior digastrics would overcontour posteriorly, and (4) transitions between the superior constrictor and medial constrictor, medial constrictor and inferior constrictor, and inferior constrictor and cricopharyngeus would be incomplete by missing a slice between structures or by splitting into islands at the transition. In general, the algorithm performed well in the presence of dental artifact (Fig. 4-A1). Examples of minor edits needing to be performed (e.g. in the presence of tumor or dental stent which distorts the tongue) are in the supplementary material.

## 4 Discussion

This work demonstrated how a publicly available deep learning tool and expertly curated data (n = 32 lymph node dataset, n = 78 swallowing/chewing structure dataset) can result in a practical segmentation tool that is both accurate and clinically acceptable. Furthermore, this work resulted in two published datasets (in The Cancer Imaging Archive) that are available for clinical researchers to reproduce or build upon the field of head and neck CT-based tissue segmentation. To our knowledge, these two datasets represent the first publicly available datasets for swallowing and chewing structures and lymph nodes contoured on non-contrast CT, both with physician scores of clinical acceptability.

One early work in lymph node and swallowing structures segmentation in the head and neck was by Teguh et al., which obtained a DSC of 0.73 on average for lymph node levels I-V and 0.50–0.71 DSC for swallowing and chewing structures [21]. While various works such as van der Veen and Weissmann et al., reported qualitative evaluations (e.g. radiation oncologist scores) and quantitative evaluations (e.g. DSC, HD) for lymph node model contours, few authors reported what proportion of deep learning contours require edits before the contours can be used clinically. For swallowing and chewing structures, most authors have reported what percentage of their test set contours require edits before clinical use. These rates have ranged from 100 % to 18 % for various structures with DSC’s ranging from 0.5 to at most 0.91 on average or median (see supplementary material). In addition to accuracy and clinical acceptability, previous works have extensively studied human interobserver variability in lymph node level contouring and have quantified it in terms of DSC [25], [26]. Weissmann et al., demonstrated that when physicians re-contoured their own volumes, DSC was 0.77 on average [26]. Overall, they reported that the accuracy of their deep learning approach performed with 0.78 DSC on average across 20 nodal levels.

Regarding the lymph nodes segmentation performance of our approach, quantitative analysis of the lymph node level segmentations indicated that DSC ranged from 0.77 to 0.89 across all levels and is comparable to other recent approaches in the literature (see supplementary material for comparison). However, it is important to consider that our quantitative results may be inflated due to bias introduced in the data curation process described in the methods section. Regarding qualitative evaluation, a blinded study investigating physician preference indicated that the AI contours were superior to or equally preferred to the manual contours at rates ranging from 75 % to 91 %. In addition, a blinded study of clinical acceptability indicated that there was no significant difference in clinical acceptability between manual contours and AI contours for levels IA, IB, II, III, IV, and V, with greater clinical acceptability demonstrated for AI-generated RP contours. Furthermore, only 8 % of AI lymph node contours require edits for clinical use as determined by our team of sub-specialized head and neck radiation oncologists. Thus, the hypothesis of this work can be accepted, since 1) there is no difference in clinical acceptability between manual and AI contours and 2) the AI contours were superior to or equally preferred to the manual contours. Although not directly comparable, our average DSC’s in the nodal test set are all greater than previously reported measures of interobserver variability mentioned above. Anecdotally, this may suggest that our model’s accuracy has similar or smaller levels of variance compared to human contouring in our practice since there was no preference for manual vs AI contours (p < 0.01) for all but one nodal level. Weissmann et al., demonstrated that a clinically acceptable lymph segmentation model could be trained with the publicly available nnUnet tool with a small cohort of patients (n = 35) [26]. Likewise, we have found this number to be adequate for our patient population of head and neck patients which receive radiotherapy simulation CTs.

Regarding the swallowing structures segmentation, our work produced a model that segments 17 different swallowing and chewing structures, more than any work, to our knowledge, has produced. Median DSC was greater than 0.86 for all structures, and although values in the literature cannot be directly compared between different datasets, our approach yields the most accurate structures reported in terms of DSC. Furthermore, it was demonstrated that 96 % percent of contours in the test set could be used clinically without edit and this percentage is much greater than any rates previously reported in the literature for swallowing and chewing structures (see supplemental material). Dental stents distorting tongue position and the presence of abutting tumor were common factors which necessitated minor edits. The hypothesis of this work, that swallowing and chewing AI contours are clinically acceptable, can be accepted.

Future studies would benefit from studying the effects of patient positioning, adenopathy, nodal level dissection, bulky disease, and presence of contrast upon segmentation performance. In this work, training and testing cohorts for both datasets used a three-step methodology in which ground truth data was generated by a sparsely trained deep learning model or atlas-based model. Although this strategy is useful for perpetuating sparse data, it can greatly bias the curation process and quantitative evaluation. This is especially true if contours are not comprehensively edited after it is generated by the atlas or sparsely trained nnUnet model (the nnUnet predictions required only minor revisions; the atlas required more extensive revisions). These models for lymph nodes and swallowing and chewing structures are being integrated into our clinical practice as well as the Radiation Planning Assistant (RPA), a web-based, FDA 510 k cleared platform that provides contouring and planning to clinics with low resources [36], [37].

In conclusion, this work demonstrated how a publicly available deep learning tool and expertly curated data can result in a practical segmentation tool that is both accurate and clinically acceptable. All testing and training data are being made publicly available on The Cancer Imaging Archive. Future work will involve multi-institutional studies to evaluate robustness as well as application based-works that use these tools for clinical trial quality assurance.

## CRediT authorship contribution statement

**Sean Maroongroge:** Data curation, Investigation, Project administration, Supervision, Writing – review & editing, Writing – original draft, Methodology, Conceptualization. **Abdallah SR Mohamed:** Writing – review & editing, Investigation, Data curation. **Callistus Nguyen:** Visualization, Writing – original draft, Writing – review & editing, Data curation, Resources, Software. **Jean Guma De la Vega:** Writing – review & editing, Visualization, Data curation. **Steven J. Frank:** Writing – review & editing, Investigation. **Adam S. Garden:** Writing – review & editing, Investigation. **Brandon G. Gunn:** Investigation, Writing – review & editing. **Anna Lee:** Investigation, Writing – review & editing. **Lauren Mayo:** Investigation, Writing – review & editing. **Amy Moreno:** Investigation, Writing – review & editing. **William H. Morrison:** Investigation, Writing – review & editing. **Jack Phan:** Investigation, Writing – review & editing. **Michael T. Spiotto:** Supervision, Writing – review & editing. **Laurence E. Court:** Supervision, Writing – review & editing. **Clifton D. Fuller:** Supervision, Writing – review & editing, Project administration. **David I. Rosenthal:** Supervision. Writing – review & editing, Investigation. **Tucker J. Netherton:** Writing – original draft, Writing – review & editing, Supervision, Visualization, Project administration, Visualization, Investigation, Validation, Software, Formal analysis.

## Declaration of competing interest

The authors declare the following financial interests/personal relationships which may be considered as potential competing interests: Dr. Fuller received/receives related funding and salary support from: National Institutes of Health (NIH) National Cancer Institute (NCI) and  National Institute of Dental and Craniofacial Research (NIDCR) Administrative Supplements to Support Collaborations to Improve AIML-Readiness of NIH-Supported Data (R01CA257814-02S3; R01DE028290-04S2); NIH National Institute of Biomedical Imaging and Bioengineering (NIBIB) Research Education Programs for Residents and Clinical Fellows Grant  (R25EB025787);  NIH/NCI Cancer Center Support Grant (CCSG) (P30CA016672);  Patient-Centered Outcomes Research Institute (PCS-1609–36195;  sub-award from Princess Margaret Hospital). Dr. Fuller receives grant and infrastructure support from MD Anderson Cancer Center via the Charles and Daneen Stiefel Center for Head and Neck Cancer Oropharyngeal Cancer Research Program. Dr. Fuller has received NIH sub-award support from  Oncospace, Inc. (R43CA254559, PI Lakshminarayanan) under a Small Business Innovation Research Grant Applications grant. Dr. Fuller has received direct industry grant/in-kind support, honoraria, and travel funding from Elekta AB. Dr. Fuller has served as a consulting capacity for Varian/Siemens Healthineers, Philips Medical Systems, and Oncospace, Inc.; Dr. Court receives funding (last 36 months) from NCI, CPRIT, Wellcome Trust,The Fund for Innovation in Cancer Informatics, Varian Medical Systems. Dr. Frank has grant funding from Hitach for a Phase II/III Randomized Head and Neck Trial. Dr. Frank is a paid consultant from IBA and Boston Scienfitic. Dr. Frank is the founder and Chair of the Scientific Advisory Committee of C4 Imaging with patent, royalty, and ownership interest. None of the financial interests or personal relationships appear to influence the work reported in this paper. The authors thank the MD Anderson High Performance Computation group and Tumor Measurement Initiative for their support and use of resources.
