# Supplementary figures and images for: Clinical acceptability of automatically generated lymph node levels and structures of deglutition and mastication for head and neck radiation therapy
Source: Phys Imaging Radiat Oncol. 2024 Feb 1;29:100540. doi: 10.1016/j.phro.2024.100540 (PMC10864833; doi:10.1016/j.phro.2024.100540)

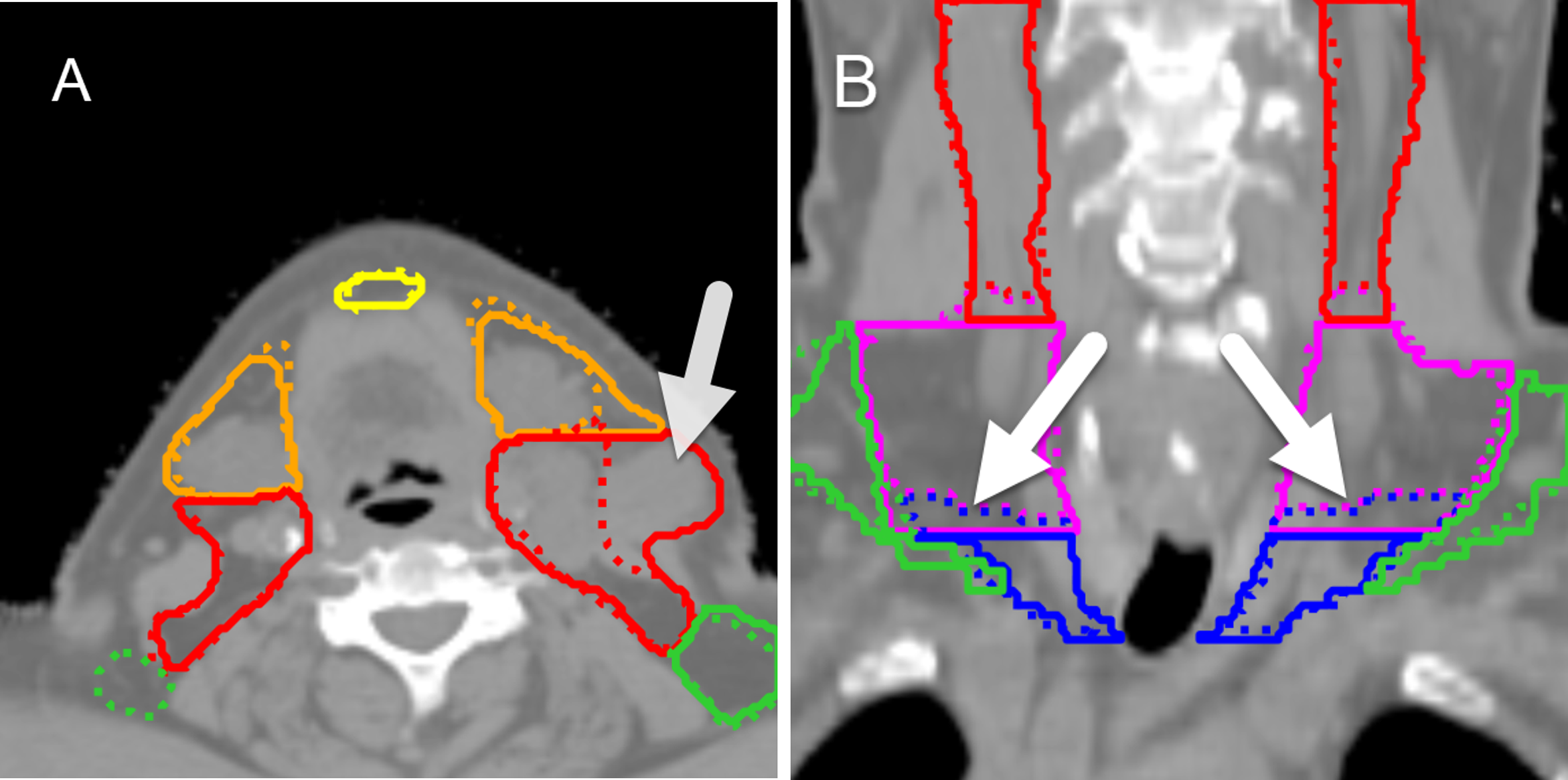

Supplement: Supplementary Data 1 [file mmc1.zip › sup1.tif]

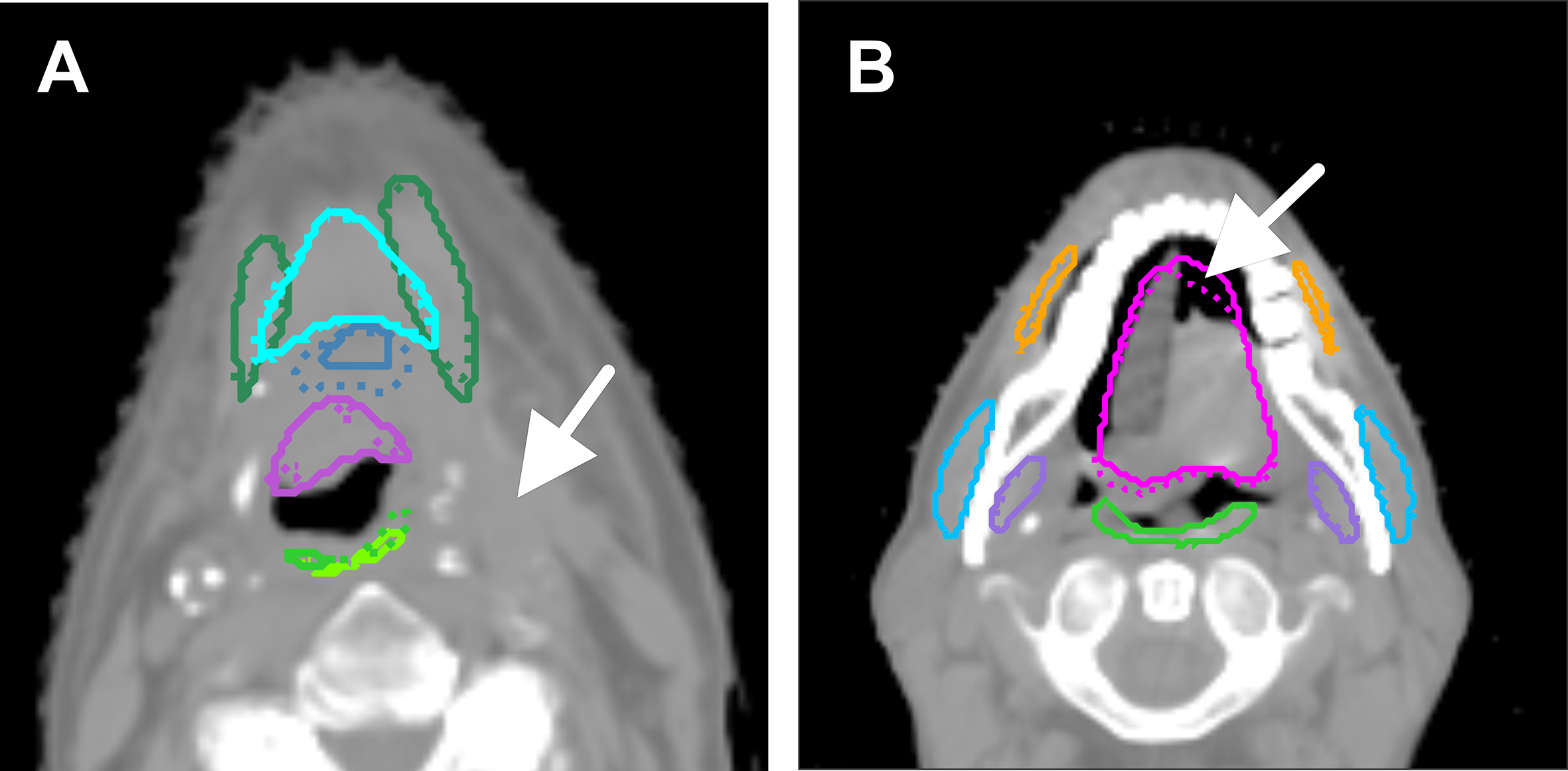

Supplement: Supplementary Data 1 [file mmc1.zip › supp2.tif]

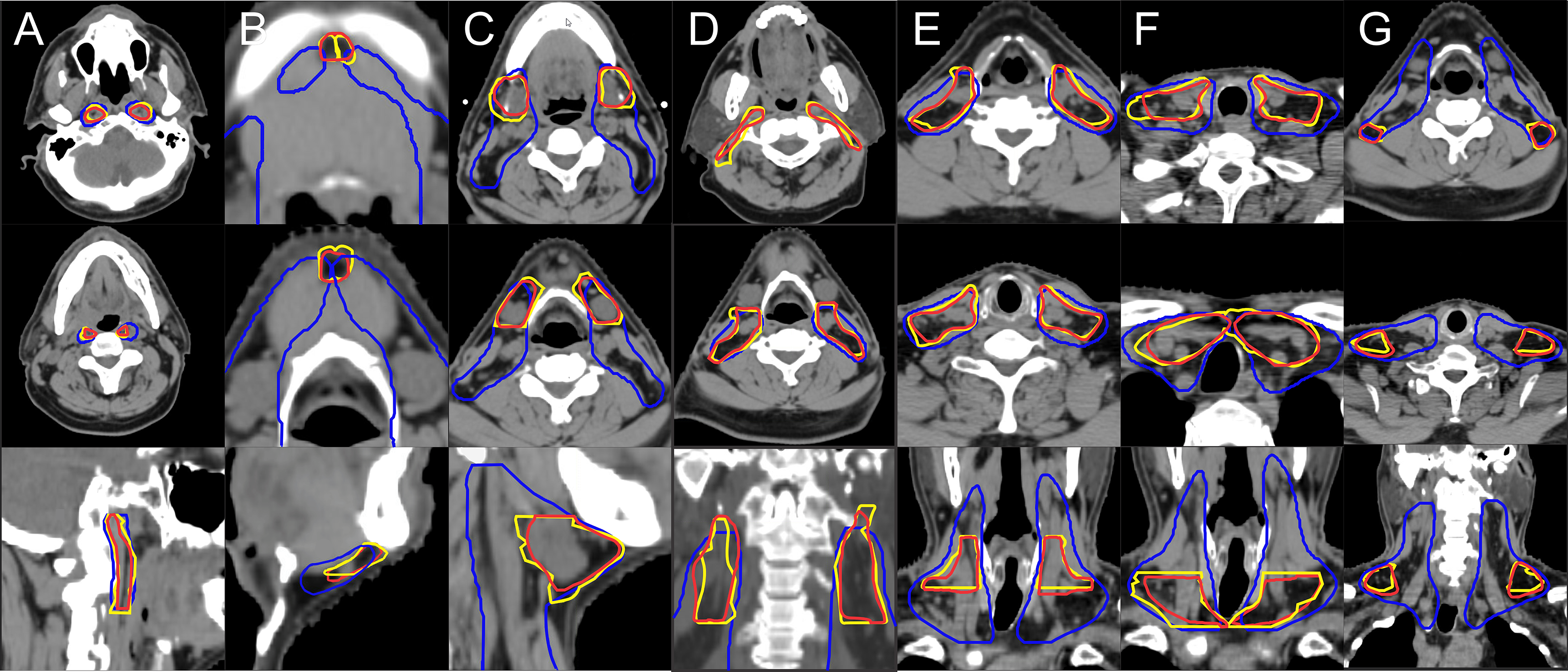

Supplement: Supplementary Data 1 [file mmc1.zip › supp3.tif]
